# Spontaneous retroperitoneal hematoma: a case report

**DOI:** 10.1186/s13256-023-03794-4

**Published:** 2023-02-28

**Authors:** Takuma Kurotaki, Naoya Okada, Yasuo Sakurai, Takumi Yamabuki, Minoru Takada, Kentaro Kato, Takeshi Yokoyama, Yoshiyasu Ambo, Yoshihiro Kinoshita, Fumitaka Nakamura, Nobuichi Kashimura

**Affiliations:** 1 Department of Surgery, Teine Keijinkai Hospital, 1-29 Maeda, Sapporo, Hokkaido 006-8555 Japan; 2 Department of Medical Radiation Technology, Teine Keijinkai Hospital, Sapporo, Hokkaido Japan; 3 Department of Anesthesiology, Teine Keijinkai Hospital, Sapporo, Hokkaido Japan

**Keywords:** Spontaneous retroperitoneal hematoma, Conservative management, Transcatheter arterial embolization, Surgical intervention, Algorithm

## Abstract

**Background:**

Spontaneous retroperitoneal hematoma is defined as bleeding in the retroperitoneal space without any triggers such as trauma, invasive procedures, and abdominal aortic aneurysm.

**Case presentation:**

A 48-year-old Japanese man who experienced sudden abdominal pain, severe hypotension, and decreased hemoglobin was diagnosed with spontaneous retroperitoneal hematoma. Contrast-enhanced computed tomography revealed massive left retroperitoneal hematoma; however, neither extravasation nor causative aneurysm was noted. Through conservative management with close monitoring, he was treated and discharged on the tenth hospital day without any morbidity.

**Conclusions:**

Spontaneous retroperitoneal hematoma treatment comprises conservative management, transcatheter arterial embolization, and surgical intervention. The mortality rate of spontaneous retroperitoneal hematoma is so high that the optimal treatment timing needs to be carefully judged on the basis of detailed evaluation, and management algorithm with clear criteria.

**Supplementary Information:**

The online version contains supplementary material available at 10.1186/s13256-023-03794-4.

## Background

Spontaneous retroperitoneal hematoma (SRH) is a potentially fatal disease that usually occurs during antiplatelet or anticoagulant therapy [1]. The treatment comprises conservative management, transcatheter arterial embolization (TAE), and surgical intervention [2]. These treatment options are selected case-by-case without guidelines. We report a case of SRH resulting in hemorrhagic shock, which was successfully treated via conservative management with close monitoring, and propose a diagnostic and management algorithm based on our experience and a review of the existing literature.

## Case presentation

**Fig. 1:**
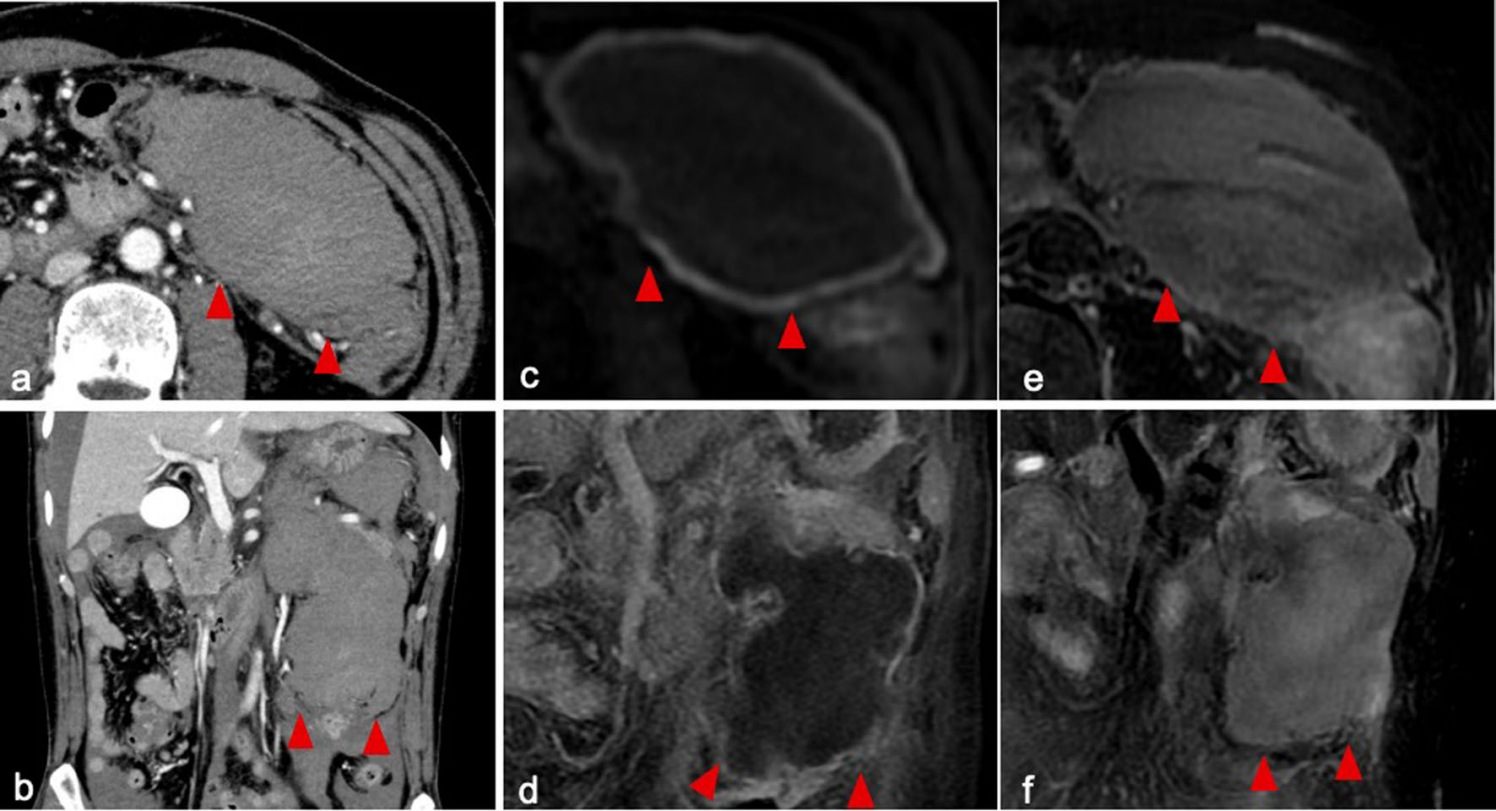
CT and MRI of the abdomen. **a** Contrast-enhanced CT revealed a large low-density area (arrow heads) in the left peritoneal space. **b** Coronal contrast-enhanced CT revealed a low-density area (arrow heads) in the left retroperitoneal space. Neither extravasation nor causative aneurysm was observed. **c** T1-weighted MRI revealed that the mass (arrow heads) was encapsulated by hyperintense rims. **d** Coronal T1-weighted MRI performed after gadolinium administration revealed no extravasation in the mass (arrowheads). **e** T2-weighted MRI showed homogeneously high intensity in the mass (arrowheads). **f** Coronal T2-weighted MRI showed homogeneously high intensity in the mass (arrowheads). *CT* computed tomography, *MRI* magnetic resonance imaging

**Fig. 2:**
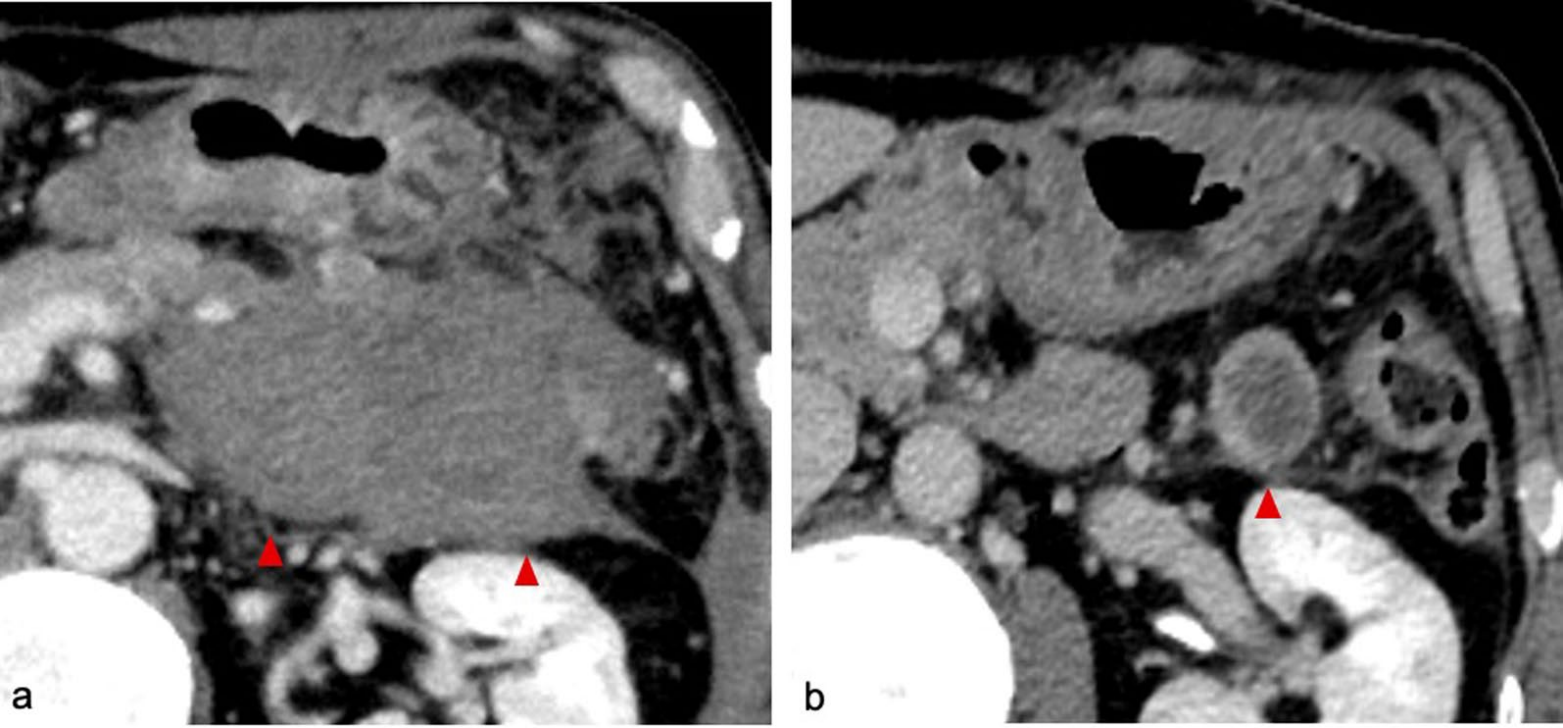
Contrast-enhanced CT of the abdomen. **a** Contrast-enhanced CT on the fifth hospital day showed a large hematoma (arrowheads) in the left peritoneal space. **b** Contrast-enhanced CT 5 months after discharge revealed that the hematoma (arrowhead) was remarkably reduced. *CT* computed tomography

A 48-year-old Japanese man was admitted to a local hospital with sudden abdominal pain. A previous medical checkup had revealed hypertension and dyslipidemia, but he had no other medical history. The patient was an office worker who had no relevant social, environmental, or family history. He did not have a prior smoking habit or history of alcohol drinking. He was not taking any medication at home, and he denied any history of trauma. Upon arrival, his systolic blood pressure was 50 mmHg, and dobutamine therapy was initiated. Computed tomography (CT) revealed a low-density area in the left upper quadrant, and gastrointestinal stromal tumor (GIST) was suspected. His hemoglobin (Hb) level was 14.2 g/dL on arrival, which decreased to 8.7 g/dL on the following day; hence, he was referred to us for further examination and treatment. Upon arrival at our hospital, he was febrile with a body temperature of 99.6 ℉, hypotensive with a blood pressure of 97/62 mmHg, and had a heart rate of 118 beats per minute. His oxygen saturation was 97% on room air. His circulation was maintained by continuous intravenous infusion of 7 μg/kg/minute of dobutamine. On physical examination, the patient was alert with a Glasgow Coma Scale score of 15, and he had no signs of neurological deficits. His abdominal examination showed a soft abdomen with tenderness upon palpation in the left upper and lower quadrants. Laboratory tests were performed, which showed a white blood cell count of 14.4 × 10^3^/μl (reference range, 3.3–8.6 × 10^3^/μl), hemoglobin concentration of 9.7 g/dl (reference range, 13.7–16.8 g/dl), and platelet count of 231 × 10^3^/μl (reference range, 158–348 × 10^3^/μl). His prothrombin time was 13 seconds (reference range, 10–13 seconds), activated partial thromboplastin time was 27.8 seconds (reference range, 26.0–38.0 seconds), and fibrinogen concentration was 433 mg/dl (reference range, 170–410 mg/dl). His total bilirubin concentration was 0.2 mg/dl (reference range, 0.4–1.5 mg/dl), aspartate aminotransferase concentration was 172 U/l (reference range, 13–30 U/l), and alanine aminotransferase concentration was 176 U/l (reference range, 10–42 U/l). His blood urea nitrogen concentration was 41.9 mg/dl (reference range, 8.0–20.0 mg/dl), and his creatinine concentration was elevated to 3.03 mg/dl (reference range, 0.65–1.07 mg/dl). His C-reactive protein concentration was 7.71 mg/dl (reference range, 0.00–0.14 mg/dl). The results of hepatitis B, hepatitis C, and human immunodeficiency virus (HIV) testing, as well as blood culture, were negative. Urinalysis was not performed. Contrast-enhanced CT revealed a massive homogeneous low-density area in the left retroperitoneum, which was suspected to be a giant hematoma (Fig. 1a, b). No extravasation, arterial aneurysm, or tumor was observed. An arterial line and a central venous catheter were introduced, and dobutamine was switched to noradrenaline 0.01 μg/kg/minute via this route. He had renal dysfunction [estimated glomerular filtration rate (eGFR): 19 ml/minute/1.73 m^2^], which was possibly due to severe dehydration and compression of the renal parenchyma and renal vessels. He did not take any anticoagulant or antiplatelet medications and his coagulation status was normal. Angiography was not performed because the contrast-enhanced CT did not reveal extravasation, and he had severe renal dysfunction. Because his circulation was maintained with fluid resuscitation, noradrenaline, and blood transfusion, conservative treatment with close monitoring in the intensive care unit (ICU) was indicated. Acetaminophen (1000 mg) was intravenously administered for pain. Noradrenaline administration was terminated on the next day. A total of 8 units of red blood cells were administered to maintain his Hb level within 7–9 g/dl. He was discharged from the ICU on the fifth day because his symptoms had resolved and the contrast-enhanced CT did not reveal hematoma growth (Fig. 2a). We prescribed amlodipine besylate at 2.5 mg/day for hypertension on the sixth day. On the seventh day, enhanced magnetic resonance imaging (MRI) revealed heterogeneous low T1 and high T2 signals (Fig. 1c–f); therefore, hematoma was confirmed. No causative bleeding tumor was observed. As his vital signs were stable, we stopped monitoring them on the same day. He was discharged on the tenth hospital day without any morbidity. In the outpatient clinic, contrast-enhanced CT performed 5 months later revealed that the hematoma was remarkably reduced (Fig. 2b). MRI performed at the same time revealed that the hematoma was localized to the retroperitoneal space and involved no other organs, suggesting that the source of the bleeding was not GIST (Additional file 1). The patient had an uneventful clinical course after hospital discharge, and he developed no recurrence for more than 2 years.

## Discussion and conclusions

We have herein presented a case of SRH with an unstable hemodynamic status that was successfully managed via conservative treatment with close monitoring. This case was unique because the patient took no anticoagulants and the SRH was resolved conservatively. Below, we suggest an evaluation criteria and management algorithm for SRH.

SRH is a relatively rare clinical entity that involves bleeding in the retroperitoneal space without any triggers. SRH usually occurs in the population taking anticoagulants [1]. However, our patient was not taking any anticoagulant or antiplatelet medications. Bleeding can occur in any retroperitoneal vessels and organs [2]. Although the pathogenesis of SRH is unclear, it may be caused by arteriosclerosis of microvessels [2], vascular lesions such as segmental arterial mediolysis [3], or unrecognized minor [2] trauma. The cause of bleeding is often unclear.

CT is a rapid and readily available test to identify retroperitoneal hematoma [4]. Extravasation is an independent predictor for TAE or surgical intervention [5]. MRI is helpful in establishing an accurate diagnosis owing to its efficient detection of retroperitoneal structures, including fat, organs, tumors, and hematoma [6]. Although MRI is not always necessary for diagnosis in the acute phase, it may be useful for differential diagnoses. Angiography is effective for detecting the hemorrhagic site and can enable the embolization of the affected arteries to stop the bleeding [7]. In the present case, contrast-enhanced CT revealed a low-density area in the central and left retroperitoneal space; however, neither extravasation nor causative aneurysm was observed. Enhanced MRI revealed heterogeneous low T1 and high T2 signals, indicating hematoma. Hence, the patient was diagnosed with SRH.

**Fig. 3:**
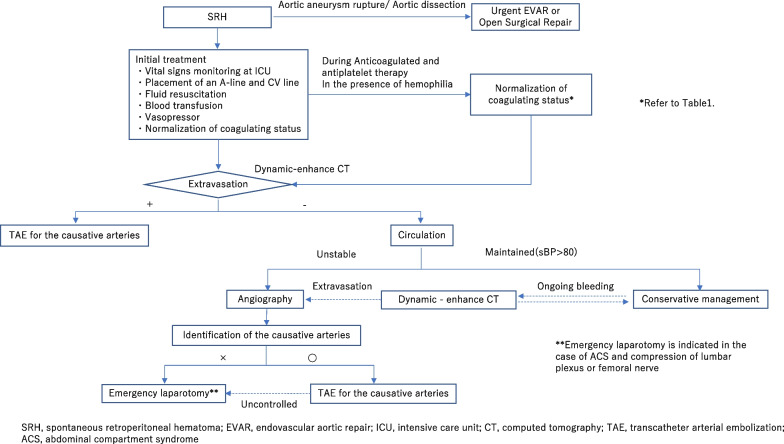
Algorithm for diagnosis and treatment of retroperitoneal hematoma. *SRH* spontaneous retroperitoneal hematoma, *EVAR* endovascular aortic repair, *ICU* intensive care unit, *CT* computed tomography, *TAE* transcatheter arterial embolization, *ACS* abdominal compartment syndrome

We present the diagnostic and management algorithm of SRH based on a literature review (Fig. 3, Table 1). If the bleeding occurs because of aortic aneurysm rupture, urgent endovascular aneurysm repair (EVAR) or open surgical repair is required [8]. Retroperitoneal hematoma can be treated conservatively when there is no persistent bleeding and the circulation is maintained, whereas endovascular or surgical treatment is required when the circulation is unstable [2]. When the circulation is stable with continuous transfusion or vasopressor infusion, ongoing bleeding is suspected [9], and dynamic enhanced CT should be performed again.

**Table 1:** Details of normalization of coagulating status

State	Normalization of coagulating status
During anticoagulated and antiplatelet therapy	Discontinue the medication Reversals such as vitamin K, idarucizumab, and PCC
Platelet count > 5 × 10^9^/l	Platelet transfusion
Clauss fibrinogen level ≤ 1.5 g/l	Administration of FFP
Presence of hemophilia	aPCC and recombinant factor VIIa

*PCC* prothrombin complex concentrate, *FFP* fresh frozen plasma, *aPCC* active prothrombin complex concentrate

Most patients with retroperitoneal hematoma take anticoagulant and/or antiplatelet medications. Discontinuation of these antithrombotic agents and the reversal agents, such as vitamin K, idarucizumab, and prothrombin complex concentrate, are recommended [10]. In the presence of congenital or acquired hemophilia, active prothrombin complex concentrate and recombinant factor VIIa should be administered [11]. The European guideline on the management of major bleeding and coagulopathy following trauma states that, in cases of hemorrhagic shock, fluid resuscitation should be started and vasopressors, including norepinephrine, can be administered to achieve systolic blood pressure of 80–90 mmHg [10]. Transfusion is required to maintain Hb level of 7–9 g/dl and platelet count above 50 × 10^9^/l [10]. The guideline also recommends treatment with fibrinogen concentrate or cryoprecipitate when Clauss fibrinogen levels are ≤ 1.5 g/l [10]. Fresh frozen plasma (FFP) administration is suggested if prothrombin time and/or activated partial thromboplastin time are > 1.5 times the normal values [10]. In this case, the patient was in hemorrhagic shock, and the bleeding seemed to be persistent; however, his blood pressure could be maintained by fluid resuscitation, vasopressor infusion, and blood transfusion. Enhanced CT did not show extravasation and the bleeding site. Angiography was not performed because the possibility of active bleeding was less likely and embolization of the causative arteries was not necessary. Conservative management with close monitoring in the ICU was chosen so that urgent TAE or laparotomy could be performed in case of prolonged and worsening shock state resulting from rebleeding. The retroperitoneal cavity is a relatively narrow space, and the tamponade effect of the hematoma reportedly stops bleeding [2]. Sunga *et al*. reported that the overall mortality of SRH within 30 days was as high as 10.1% [1]. Because of the high mortality rate, SRH cases need to be monitored carefully.

Tani *et al*. reported that, in a retrospective study of 20 consecutive cases of SRH, TAE could achieve hemostasis in 17 out of 19 patients [7]. In four of these patients, the causative arteries could not be identified, and the arteries around the hematoma were embolized empirically [7]. Even if the origin of bleeding is not detected, empirical embolization might stop life-threatening bleeding. In this case, angiography was not performed because the patient was hemodynamically stable following fluid resuscitation and vasopressor infusion, and had significant renal dysfunction due to dehydration.

Surgical treatment is performed to stop the bleeding and remove the hematoma [2]. Emergency laparotomy is indicated in cases of abdominal compartment syndrome, and compression of the lumbar plexus or femoral nerve [12]. In a report of cases in which surgery was performed prior to TAE, the bleeding site was not intraoperatively identified in all of the four patients [13]. If the bleeding site cannot be identified, intra-abdominal packing can stop the retroperitoneal bleeding, and reoperation can be planned after 48 hours [10] for reexamining whether hemostasis is achieved [2]. In the hemodynamically unstable emergency cases, TAE should be performed in a hybrid operating room, and if the bleeding cannot be stopped by TAE, emergency laparotomy is needed.

We report a rare case of SRH with an unstable hemodynamic status that was successfully managed via conservative treatment with close monitoring. As the mortality rate of SRH is high, SRH management requires systematic and close monitoring based on our suggested clear evaluation criteria and management algorithm.

## Supplementary Information

**Additional file 1. **MRI after discharge. **A** T1-weighted MRI 5 months after discharge revealed that the high-intensity area was localized to the retroperitoneal space and involved no other organs, suggesting that the source of the bleeding was not GIST. **B** T2-weighted MRI 5 months after discharge revealed that the low-intensity area was localized to the retroperitoneal space and involved no other organs. *CT* computed tomography, *MRI* magnetic resonance imaging, *GIST* gastrointestinal stromal tumor.

## Data Availability

Not applicable.

## Abbreviations

SRH: Spontaneous retroperitoneal hematoma
TAE: Transcatheter arterial embolization
CT: Computed tomography
GIST: Gastrointestinal stromal tumor
Hb: Hemoglobin
ICU: Intensive care unit
MRI: Magnetic resonance image
EVAR: Endovascular aneurysm repair
FFP: Fresh frozen plasma
