# Supplementary figures and images for: Spontaneous retroperitoneal hematoma: a case report
Source: J Med Case Rep. 2023 Feb 28;17:70. doi: 10.1186/s13256-023-03794-4 (PMC9972597; doi:10.1186/s13256-023-03794-4)

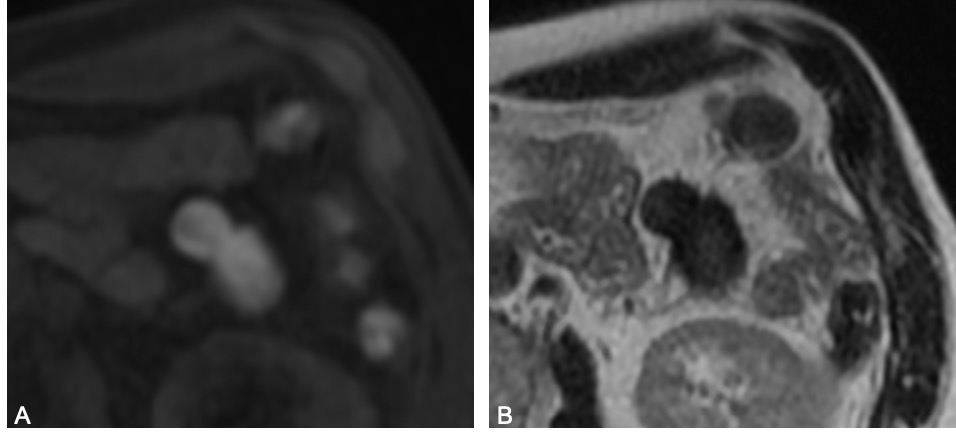

Supplement: Supplementary file 1 — Additional file 1. MRI after discharge. A T1-weighted MRI 5 months after discharge revealed that the high-intensity area was localized to the retroperitoneal space and involved no other organs, suggesting that the source of the bleeding was not GIST. B T2-weighted MRI 5 months after discharge revealed that the low-intensity area was localized to the retroperitoneal space and involved no other organs. CT computed tomography, MRI magnetic resonance imaging, GIST gastrointestinal stromal tumor. [file 13256_2023_3794_MOESM1_ESM.jpg]
